# Draft genomic sequences of *Vibrio cholerae* strains linked to the cholera outbreak in Kamituga, South Kivu, DRC

**DOI:** 10.1128/mra.01044-24

**Published:** 2025-03-20

**Authors:** Pimlapas Leekitcharoenphon, Pacifique Ndishimye, Saria Otani, Frederik Duus Møller, Leonard Schuele, Bas B. Oude Munnink, Jean Claude Udahemuka, Freddy Belesi Siangoli, Justin Bengehya Mbiribindi, Marion Koopmans, Frank M. Aarestrup, Leandre Murhula Masirika

**Affiliations:** 1 Research Group for Genomic Epidemiology, National Food Institute, Technical University of Denmark, Kgs. Lyngby, Denmark; 2 Genomics Research and Development Division, Stansile Research Organization, Kigali, Rwanda; 3 Department of Viroscience, Erasmus University Medical Center, Rotterdam, the Netherlands; 4 Department of Veterinary Medicine, University of Rwanda, Nyagatare, Rwanda; 5 Division Provinciale de la Santé, Bukavu, South-Kivu, Democratic Republic of Congo; 6 Centre de Recherche en Sciences Naturelles de Lwiro, DS Bukavu, South Kivu, Democratic Republic of Congo; 7 SaBio Instituto de Investigación en Recursos Cinegéticos IREC (Universidad de Castilla-La Mancha & CSIC), Ciudad Real, Spain; 8 Congo Outbreaks, Research for Development, Bukavu, South Kivu, Democratic Republic of Congo; Loyola University Chicago, Chicago, Illinois, USA

**Keywords:** *Vibrio cholerae*, South Kivu, DRC

## Abstract

*Vibrio cholerae* is responsible for outbreaks in Africa, but the cause of the outbreaks remains poorly understood. Here, we report the draft genomes of four *Vibrio cholerae* strains isolated from individuals affected by an outbreak in Kamituga, South Kivu, the Democratic Republic of the Congo, between January and May 2024.

## ANNOUNCEMENT

*Vibrio cholerae* presents a significant public health challenge globally, particularly in regions with inadequate water and sanitation infrastructure. *V. cholerae* causes severe diarrhea, which can lead to death if left untreated (1). In Africa, cholera is endemic in several countries, and outbreaks are frequent. The Democratic Republic of the Congo (DRC) is among the countries with a high burden of cholera and frequent outbreaks (2). The recent cholera outbreak in 2023 in the DRC has been particularly severe, with thousands of cases reported across multiple provinces (3).

Understanding the genomic characteristics of *V. cholerae* strains circulating in the DRC is crucial for effective outbreak control and the development of interventions. Here, we report the genome sequences of four *V. cholerae* strains isolated from human fecal samples in outbreak incidents in Kamituga during January to May 2024 (Table 1). The genomes could enhance our understanding of the epidemiology of cholera in the wider region and contribute to the efforts for controlling cholera outbreaks.

**TABLE 1:** Characteristics of the four *Vibrio cholerae* strains from DRC

Strain ID	Country	Month and year	ST type	Serotype	Sample source
VC2	DRC	February 2024	ST69	O1/El Tor Variant	Fecal
VC4	DRC	February 2024	ST69	O1/El Tor Variant	Fecal
VC9	DRC	February 2024	ST69	O1/El Tor Variant	Fecal
VC6	DRC	May 2024	ST69	O1/El Tor Variant	Fecal

Strain ID	Sulfamethoxazole	Trimethoprim	Chloramphenicol	Streptomycin	Florfenicol
VC2	*sul2*	*dfrA1*	*^a^-*	*-*	*-*
VC4	*-*	*dfrA1*	*catB9*	*-*	*-*
VC9	*sul2*	*dfrA1*	*catB9*	*aph (6)-Id*	*floR*
VC6	*sul2*	*dfrA1*	*catB9*	*aph (6)-Id*	*floR*

Strain ID	No. of contigs	Total length (bp)	No. of reads	Coverage (X)	GC content (%)	N50 raw reads	N50 assembly	GenBank ID	ENA run accession
VC2	15	4,063,035	60,6511	146.812	47.52	1,926	698545	GCA_041449115.1	ERR13511847
VC4	12	4,035,589	463,057	111.279	47.46	1,668	510713	GCA_041413065.1	ERR13511848
VC9	2	4,097,011	1,054,868	174.288	47.50	814	3056088	GCA_041937545.1	ERR13511852
VC6	2	4,098,385	469,720	390.195	47.51	6,196	3057686	GCA_041413135.1	ERR13511850

^*a*^ “*-*”, Not detected.

The *V. cholerae* strains were selectively isolated from fecal samples on the thiosulfate–citrate–bile salts–sucrose (TCBS) agar inoculated in alkaline peptone water with 37 degree Celsius and 24 hours for enrichment. DNA was extracted from pure colonies in the TCBS media using the DNeasy Blood & Tissue Kit (Qiagen). The DNA was sequenced on the Oxford Nanopore Technology platform Mk1B for long reads using the ligation library protocol with 14 chemistry (SQK-NBD114.24) following the manufacturer’s instructions, without fragmentation or size selection steps (Oxford Nanopore Technology, UK). The reads were basecalled using Dorado Basecall Server v7.2.13 and the “high-accuracy model” (Table 1). Only high-quality reads (>10 Phred score) were selected, and all adapters and barcodes were trimmed prior to further analysis using MinKNOW (June 2024 version). The long raw reads were assembled using Flye version 2.9.1 (4). Subsequently, the assembly was carried out for small-scale errors such as single-base pair substitutions and indels using Medaka version 1.11.2 (5) (model r1041_e82_400bps_fast_g615) (Table 1). Assemblies from VC2, VC4, and VC9 were subjected to a binning process using MetaBAT2 version 2.12.1 (6) to retrieve only pure and high completeness of *V. cholerae* genomes. ST-type and serotype were identified using MLST Finder version 2.0.9 (7) and CholeraeFinder 1.0 (8), respectively. Antimicrobial resistance (AMR) was determined using ResFinder version 4.6.0 (9). The genomes were annotated using PGAP version 6.8 (10) and submitted to the NCBI (Table 1). The genomes were ST69 with O1 and El Tor Variant. The AMR profile can be found in Table 1. Default settings were used for bioinformatics tools. The ethical clearance to conduct these studies was obtained from the Ethical Review Committee of the Catholic University of Bukavu (number UCB/CIES/NC/022/2023).

**Fig 1:**
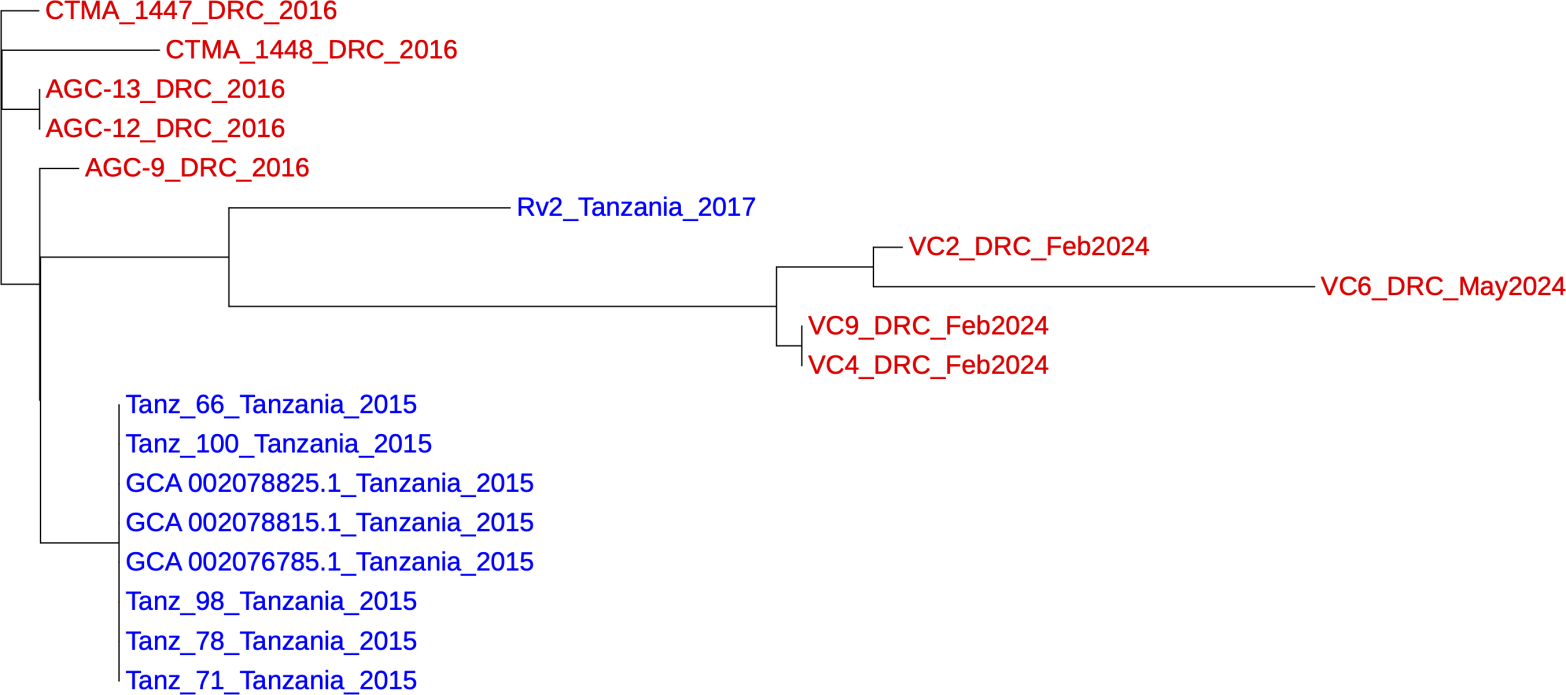
Phylogenetic tree based on core SNPs. The red labels are DRC isolates and blue are Tanzanian isolates.

The four *V. cholerae* genomes were compared with 5,991 available *V. cholerae* genomes from pathogen watch (accessed on 8th June 2024) (11) using CSI Phylogeny version 1.4 (12). Only the public genomes close to the four *V. cholerae* were included in the SNP tree in Fig. 1. This tree was constructed from 44 total core SNPs with 94% reference genome coverage (reference genome *V. cholerae* N16961, accession no. NZ_CP028827.1). The four strains from DRC were clustered together with a difference of 0–14 SNPs. Interestingly, the closest strains to the four *V. cholerae* were from Tanzania (Rv2) in 2017 with a difference of 15–27 SNPs. Another set of closer Tanzanian isolates (GCA numbers) were from Kigoma in 2015 with a difference of 17–29 SNPs.

## Data Availability

The genomic data (assemblies) were submitted in GenBank under BioProject accession number PRJNA1148388. The Oxford Nanopore reads can be downloaded at the European Nucleotide Archive (ENA) under project number PRJEB79129. Individual accession numbers can be found in Table 1.
